# Avian haemosporidians of breeding birds in the Davis Mountains sky-islands of west Texas, USA

**DOI:** 10.1017/S0031182023001087

**Published:** 2023-12

**Authors:** Viridiana Martinez, Katrina D. Keith, Jacquelyn K. Grace, Gary Voelker

**Affiliations:** Department of Ecology and Conservation Biology, Texas A&M University, College Station, TX, USA

**Keywords:** avian malaria, haemosporidians, host–parasite relationships, sky islands, west Texas

## Abstract

Avian haemosporidians are protozoan parasites transmitted by insect vectors that infect birds worldwide, negatively impacting avian fitness and survival. However, the majority of haemosporidian diversity remains undescribed. Quantifying this diversity is critical to determining parasite–host relationships and host-switching potentials of parasite lineages as climate change induces both host and vector range shifts. In this study, we conducted a community survey of avian haemosporidians found in breeding birds on the Davis Mountains sky islands in west Texas, USA. We determined parasite abundance and host associations and compared our results to data from nearby regions. A total of 265 birds were screened and infections were detected in 108 birds (40.8%). Most positive infections were identified as *Haemoproteus* (36.2%), followed by *Plasmodium* (6.8%) and *Leucocytozoon* (0.8%). A total of 71 haemosporidian lineages were detected of which 39 were previously undescribed. We found that regional similarity influenced shared lineages, as a higher number of lineages were shared with avian communities in the sky islands of New Mexico compared to south Texas, the Texas Gulf Coast and central Mexico. We found that migratory status of avian host did not influence parasite prevalence, but that host phylogeny is likely an important driver.

## Graphical abstract

**Figure:**
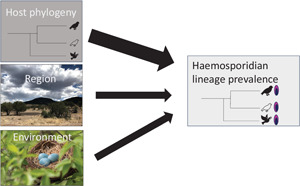

## Introduction

Avian haemosporidians (*Haemoproteus*, *Leucocytozoon* and *Plasmodium*) are intracellular protozoan parasites that infect birds worldwide (Atkinson and Van Riper, 1991; Valkiūnas, 2004; LaPointe *et al*., 2012; Clark *et al*., 2014). These parasites can impact the avian host's body condition (Dawson and Bortolotti, 2000; Garvin *et al*., 2006), survival (Marzal *et al*., 2008; Martínez-De La Puente *et al*., 2010; Van Oers *et al*., 2010; Lachish *et al*., 2011), reproduction (Asghar *et al*., 2011; Podmokła *et al*., 2014) and migration (Møller *et al*., 2004; Hegemann *et al*., 2018). However, it is estimated that the vast majority of avian haemosporidian diversity remains undescribed, especially for understudied geographic regions (Marroquin-Flores *et al*., 2017). Descriptions of these currently unknown haemosporidian communities are vital to understanding coevolutionary dynamics, biogeography and ecological niches of these abundant and highly influential parasites (Marroquin-Flores *et al*., 2017).

Various abiotic factors influence avian haemosporidian parasite prevalence, including elevation (Illera *et al*., 2017; Williamson *et al*., 2019; Gupta *et al*., 2020; Pellegrino *et al*., 2021; Lau *et al*., 2022), temperature (Pérez-Rodríguez *et al*., 2013; Ciota *et al*., 2014; Harvey and Voelker, 2017; Ishtiaq, 2021), season (Cornelius *et al*., 2014; Ham-Dueñas *et al*., 2017; Garcia-Longoria *et al*., 2019), latitude (Clark *et al*., 2020) and water availability (Wood *et al*., 2007; Krama *et al*., 2015; Sehgal, 2015; Harvey and Voelker, 2017). Climate change is predicted to influence both avian and invertebrate hosts (Brooks and Hoberg, 2007) by altering precipitation and temperature trends at global, regional and local scales (Archer and Predick, 2008; Diffenbaugh *et al*., 2008; Diffenbaugh and Giorgi, 2012). Investigations of host–parasite dynamics, especially in understudied regions, provide necessary information to determine host relationships and host-switching potential of parasite lineages, which are critical for development of effective wildlife management plans (Marroquin-Flores *et al*., 2017).

One such understudied region that is projected to experience rapid and extreme changes in climate is west Texas, where the Davis Mountains sky islands are located (Diffenbaugh and Giorgi, 2012). The Davis Mountains are isolated from other mountains by the Chihuahuan desert and rise to ~2500 m in elevation. These sky-island mountains experience a cool-temperate climate subject to summer monsoons, with cooler temperatures and increased precipitation at higher elevations (Keeling, 2017). Sky islands tend to have greater species richness due to their isolation from similar terrains (Warshall, 1995). For example, community level surveys of breeding birds in the sky islands of New Mexico, a region similar in climate to the Davis Mountains, have found a large number of novel haemosporidian lineages compared to other surveys within the United States (Marroquin-Flores *et al*., 2017; Williamson *et al*., 2019; Barrow *et al*., 2021). The Davis Mountains are also a temperate breeding ground for migrating birds travelling along the western edge of the Central Flyway of North America.

Migration can result in an increased infection risk because migrants pass through diverse habitats with differing parasites and parasite communities (Teitelbaum *et al*., 2018; Poulin and de Angeli Dutra, 2021). Migrants are an essential part of parasite dispersal by facilitating the transport of parasites from one geographic region to another (Bauer and Hoye, 2014; Poulin and de Angeli Dutra, 2021; de Angeli Dutra *et al*., 2021*a*, 2021*b*). Thus, host–parasite dynamics can differ between migrant and resident species within a single geographic region. While some studies of host–parasite relationships have found migrants to have greater parasite prevalence and richness as compared to sedentary residents species (Jenkins *et al*., 2012; Oakgrove *et al*., 2014; Walther *et al*., 2016; Poulin and de Angeli Dutra, 2021; de Angeli Dutra *et al*., 2021*a*, 2021*b*), others have found either no difference (Astudillo *et al*., 2013; Ricklefs *et al*., 2017) or higher prevalence in sedentary birds (Pellegrino *et al*., 2021). However, residents have stronger associations with their haemosporidian parasites than their migrant counterparts (Jenkins *et al*., 2012), resulting in higher associations of specialists (i.e. restricted to a single host species, or a small number of closely related host species) in residents than migrants (Hellgren *et al*., 2007). Understanding the current distribution of parasite species across host taxa (i.e. ‘host breadth’), may give an indication of future host breadth and geographic range with projected climate change (Colwell *et al*., 2008; Chen *et al*., 2011).

**Figure 1.**
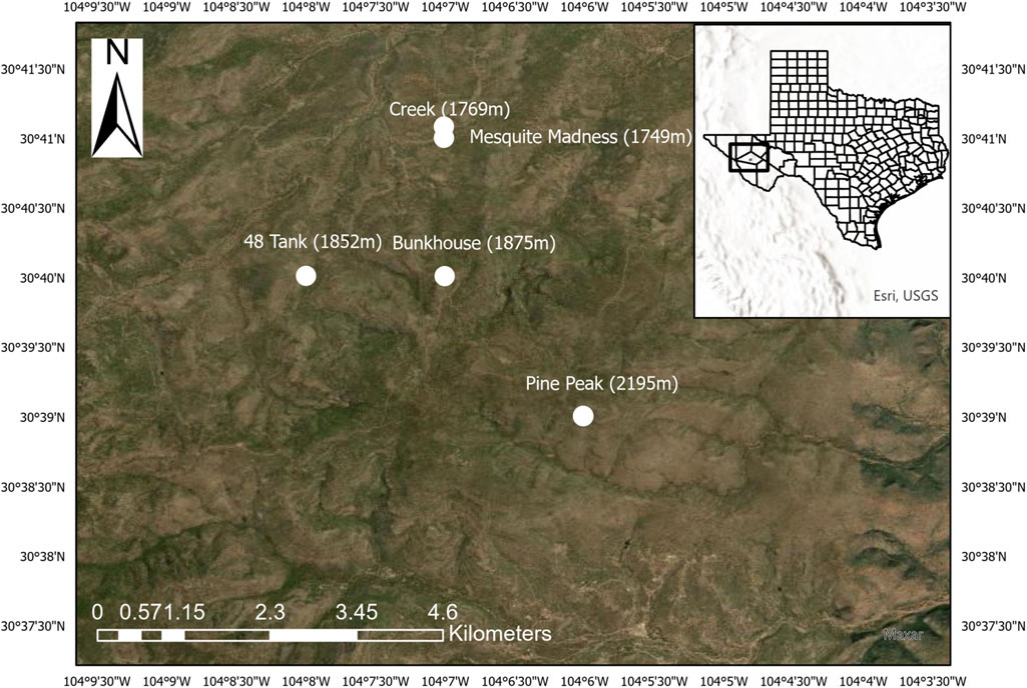
Map of sampling sites on the Davis Mountains Preserve in west Texas in 2019 and 2021.

In this study we investigated the prevalence of haemosporidian infections in birds sampled within the Davis Mountains (Fig. 1). By restricting our sampling to the breeding season (May–August) we were able to assess infections in resident species and migratory species (i.e. only present in the Davis Mountains during breeding). We reported novel lineages in this understudied region and compared infection prevalence between migrant and resident species. We also compared our results to other haemosporidian community surveys on both sky islands and non-sky islands from the region. We hypothesized that we would detect (1) *Haemoproteus* as the most abundant genus due to its high global prevalence (Hellgren *et al*., 2007; Clark *et al*., 2014); (2) higher parasite prevalence in migratory species; (3) similar prevalence rates and lineages to those detected in the sky islands of New Mexico; (4) a higher proportion of specialist lineages than generalist lineages in residents due to potential geographic isolation in the region; (5) a high number of novel lineages due to limited sampling in the region; and (6) a higher proportion of novel lineages in resident species than migrant species.

## Materials and methods

### Study site and field sampling

The Davis Mountains Preserve is 13,292 ha of protected land owned by The Nature Conservancy (TNC) in Jeff Davis County, Texas, USA (Fig. 1). We captured birds using mist nets during the breeding season (May–August) in 2019 (*n* = 121) and 2021 (*n* = 144) within an elevation band of (1746–2195 m) across 5 sites within the preserve. Sampling on the Davis Mountains Preserve occurred at 5 sampling locations, 48 Tank (1852 m elevation), Bunkhouse (1875 m), Creek (1769 m), Mesquite Madness (1746 m) and Pine Peak Pond (2195 m). The longest distance between sites was between Pine Peak Pond and the Creek which spanned 4.64 km. The shortest distance between sites was 0.61 km between the Creek and Mesquite Madness sampling sites. However, most of the samples were collected at 48 Tank and Pine Peak Pond, which were separated by 3.27 km. All sample collection sites were pinion–juniper woodland. Birds were categorized as residents (present year-round), or as migrants (present only during the breeding season). Migrant birds sampled are known to winter in Mexico, Central America, South American and the Caribbean.

Blood samples were collected in non-heparinized capillary tubes and immediately transferred into 1.5 mL Eppendorf tubes containing Queen's lysis buffer (Seutin *et al*., 1991), stored at room temperature, and transported to Texas A&M University for analysis.

### Genetic analysis on cytochrome-*b*

DNA was obtained following extraction using a E.N.Z.A. Tissue DNA Kit [Omega Bio-Tek, Norcross, Georgia, USA], following the manufacturer's protocol. Polymerase chain reaction (PCR) was used to amplify a 479 base pair portion of the haemosporidian mitochondrial cytochrome-*b* (cytb) gene using 3 primers, for each DNA sample. Each PCR used the same forward primer UNIVF and one of 3 reverse primers: UNIVR1, UNIVR2 and UNIVR3 (Drovetski *et al*., 2014). Each PCR used a positive and negative control. If a sample was negative in the initial specific primer-pair screening, it was screened a second time to confirm that result. The PCR protocol was the same for UNIVR1 and UNIVR2. Initial denaturation was for 2 min at 94°C, followed by 41 cycles of denaturation at 94°C for 30 s, annealing at 49 for 30 s, and extension at 72°C for 35 s. The PCR cycle ended with a final extension at 72°C for 5 min. UNIVR3 followed the same PCR protocol as UNIVR1 and UNIVR2 except for an annealing temperature of 49.5°C for 30 s.

PCR reactions were visualized by running 3 μL of the final PCR product on a 1% agarose gel, and positive amplifications were sequenced. These samples were purified using ExoSAP-IT [Thermo Fisher Scientific, Waltham, MA], following the manufacturer's protocol. Sanger sequencing was performed by Psomagen, USA [Rockville, MD]. Multiple infections were phased using DnasP v6 (Rozas *et al*., 2017) in order to reconstruct single infection haplotypes. These reconstructions were hereafter treated as individual infections. Parasite sequences were identified to genus using the National Library of Medicine Nucleotide BLAST tool (https://blast.ncbi.nlm.nih.gov/Blast.cgi) and to the most similar lineages using the MalAvi BLAST tool (http://130.235.244.92/Malavi/). Sequences with 100% MalAvi BLAST match were labelled as that lineage. We characterized lineages differing by 1 or more base pairs from published sequences as novel (Outlaw and Ricklefs, 2014).

We followed a standard suite of phylogenetic methods as outlined in Keith *et al*. (2022). Appropriate model selection for this dataset was performed using jModeltest 2.1.10 using BIC. Bayesian phylogenetic analyses were performed using the CIPRES Science Gateway (Miller *et al*., 2010) using Mr.Bayes 3.2.6 (Ronquist *et al*., 2012). Bayesian analysis consisted of 2 simultaneous runs for 10 million generations with 8 heated chains and sampling occurred every 1000 generations with a 25% burn-in. Convergence for each independent run was assessed using Tracer v1.7.2. Then a 50% majority rule consensus tree was constructed in FigTree v 1.4.4.

### Prevalence analysis

All statistical analyses were performed in R (R Core Team, 2020). We conducted a Fisher's exact test to investigate whether the prevalence of novel lineages differed between resident and migratory bird hosts. A Fisher's exact test was used to investigate the associations between age, sex, elevation, and foraging height of avian hosts, and haemosporidian infection prevalence. A Fisher's exact test was also used to determine whether migratory status of avian host was associated with host breadth of parasites.

Foraging height of each bird species was categorized as ground or non-ground (Billerman *et al*., 2022). Host breadth was determined for all previously described parasite lineages using the MalAvi database. Specialized parasites were determined to be those that primarily parasitize a single host species but could be detected in a single or few individuals in other host species (Drovetski *et al*., 2014). Region and host taxa of previously described lineages were compared to host taxa of the detected lineages in this study. Additionally, we compared lineages of our study to lineages detected in 5 studies across Texas, New Mexico and central Mexico (Ham-Dueñas *et al*., 2017; Marroquin-Flores *et al*., 2017; Barrow *et al*., 2021; DeBrock *et al*., 2021; Keith *et al*., 2022), whose lineages are available on the Malavi database.

## Results

### Overall prevalence

We sampled 265 individuals from 39 species and 19 families during the breeding season. Individuals of resident species comprised 47.2% (*n* = 125) of our dataset, and migratory individuals made up 52.8% (*n* = 140) of our dataset (Table 1). Overall, 108 birds (40.8%) tested positive for haemosporidian infection. Of these positive infections, *Haemoproteus* was found in 96 individuals (88.9%) (Table 2), 19 of which were co-infected by multiple *Haemoproteus* lineages. *Plasmodium* was detected in 18 birds (16.7%), 6 of which were co-infected with *Haemoproteus*. Lastly, 2 individuals (2%) were found to be co-infected with *Leucocytozoon* and *Haemoproteus.* Of the 2 main sampling locations in the Davis Mountains Preserve, 48 Tank (*n* = 80; 1852 m elevation) and Pine Peak Pond (*n* = 171; 2195 m elevation), had an overall infection prevalence of 47.5 and 37.4%, respectively (Table 2). The main genus at both sampling sites was *Haemoproteus* with a detection rate of 89.5 and 89% respectively (Table 2). A series of Fisher's exact tests determined that elevation had no effect on infection prevalence (*P* = 0.38), and age (*P* = 0.03) but not sex (*P* = 0.40) was associated with infection status. Foraging height did not influence overall haemosporidian infection rate (*P* = 0.8).

**Table 1.** Prevalence and detection rates of haemosporidian genera based on migratory status

Number				Detection rate of positive infection			
Migratory status	Total	Positive	Negative	Overall	*Haemoproteus*	*Leucocytozoon*	*Plasmodium*
Migrant	140	63	77	45%	94%	3%	11%
Resident	125	45	80	36%	82%	0%	24%

Detection rate of positive infection includes co-infections with different genera.

**Table 2.** Prevalence and detection rates of haemosporidian genera from sampling sites in the Davis Mountains Preserve

Number				Detection rate of positive infection			
Location	Total	Positive	Negative	Overall	*Haemoproteus*	*Leucocytozoon*	*Plasmodium*
48 Tank (1852 m)	80	38	42	47.5%	89.5%	0%	15.8%
Bunkhouse (1876 m)	2	0	2	0%	0%	0%	0%
Creek (1769 m)	9	4	5	44.4%	100%	0%	0%
Mesquite Madness (1747 m)	3	2	1	66.7%	50%	0%	50%
Pine Peak Pond (2195 m)	171	64	107	37.4%	89%	3.1%	17.2%
Combined Sites	265	108	157	40.8%	88.9%	1.9%	16.7%

Detection rate of positive infection includes co-infections with different genera.

A total of 45% (*n* = 63) of migratory species and 36% (*n* = 45) of residents were positive for haemosporidian infection. However, a Fisher's exact test indicated that migratory status did not affect the likelihood of haemosporidian infection (*P* = 0.17). *Haemoproteus* made up 94% (*n* = 59) and 82% (*n* = 37) of migrant and resident infections, respectively. *Plasmodium* infections were higher in residents (24%, *n* = 11) compared to migrants (11%, *n* = 7) (Table 1). Migrants were found to be infected with more generalist lineages (*n* = 15) than residents (*n* = 10), while similar numbers of specialist lineages were detected in migrants (*n* = 5) and residents (*n* = 4). A Fisher's exact test found that there was no association between host breadth and migratory status (*P* = 0.2).

### Lineage analysis

Of the 71 lineages recovered, 32 were previously described (100% BLAST matches in MalAvi) and 39 were novel lineages (differed by at least 1 base pair). The most common *Haemoproteus* lineage and the most common lineage overall was PHEMEL02, representing 19% of *Haemoproteu*s infections and 16% of all infections. The most common *Plasmodium* lineage was SETCOR03 representing 42.1% of *Plasmodium* infections and 5.4% of all infections. Of the previously described lineages, 20 were found in migrants and 16 were found in resident species. Within migrants, 16 of the previously described lineages were *Haemoproteus*, 1 was *Leucocytozoon*, and 3 were *Plasmodium*. One lineage detected in a migrant species, MYMAC02, had previously only been detected in South America (Brazil). Another lineage detected in a resident species, EULNIG01 had previously only been detected in Papua New Guinea. Within resident species, 10 of the previously described lineages were *Haemoproteus* and 6 were *Plasmodium* (Table 2).

Lineages were considered novel if there was a difference of at least 1 base pair or a similarity of less than or equal to 99% with lineages in the MalAvi database (Outlaw and Ricklefs, 2014). Based on this definition, we detected 39 novel lineages, of which 35 lineages (89.7%) were *Haemoproteus*, 3 (7.7%) were *Plasmodium* and 1 (2.6%) was *Leucocytozoon* (Tables 3 and 4). All novel lineages were only found in 1 species except for PIRLUD16 which was detected in a western tanager and a hepatic tanager; PIRFLA18 which was detected in 1 western tanager and 1 hepatic tanager and PIRFLA20 which was detected in a western tanager, hepatic tanager and willow flycatcher. Of the novel lineages detected, 26 were found in migrant and 13 were found in resident species (Table 4). Within migrant novel lineages, 23 were *Haemoproteus*, 1 was *Leucocytozoon*, and 2 were *Plasmodium*. Within resident novel lineages, 12 were *Haemoproteus*, and 1 was *Plasmodium*. Although a higher number of novel lineages were found in migrant species, a Fisher's exact test indicates that migration status did not influence the presence of novel lineages (*P* = 0.1). Sequences have been deposited on GenBank under accession numbers OR760306 - OR760450.

**Table 3.** Number of lineages recovered by host migratory status (# of previously known lineages/# of novel lineages)

Lineage	Migrant	Resident	Total
*Haemoproteus*	17/26	10/12	27/38 (65)
*Leucocytozoon*	1/1	0	1/1 (2)
*Plasmodium*	3/2	6/1	9/3 (12)

Total account for potentially the same lineage being found in both migrant and resident individuals.

**Table 4.** Haemosporidian lineages recovered, relative to avian host, migratory status and sampling location; multiple individuals of the same host species are indicated in parentheses after host name

	Malavi BLAST	% Match	BP difference	Novel lineage designation	Migratory status	Elevation
*Haemoproteus*						
Chipping sparrow (*Spizella passerina*)	(CARCAR10)	96	20	SPIPAS17 (GenBank OR760368)	Resident	H
Warbling vireo (*Vireo gilvus*)	CATUST16	100	0		Migrant	H
Lark sparrow (*Chondestes grammacus*) (7)	CHOGRA01	100	0		Resident	L, M
Lark sparrow (*Chondestes grammacus*)	(CHOGRA01)	97	13	CHOGRA03 (GenBank OR760412)	Resident	M
Chipping sparrow (*Spizella passerina*) (6)	DUNNO01	100	0		Resident	L, M, H
Black-chinned sparrow (*Spizella atrogularis*)	(DUNNO01)	99	1	SPIATR01 (GenBank OR760435)	Resident	H
Western tanager (*Piranga ludoviciana*)	(GRBRU01)	99	5	PIRLUD10 (GenBank OR760363)	Migrant	H
Woodhouse's scrubjay (*Aphelocoma woodhouseii*) (3)	GYMCYA02	100	0		Resident	L, M, H
Willow flycatcher (*Empidonax traillii*)	GYMCYA02	100	0		Migrant	H
Cassin's kingbird (*Tyrannus vociferans*)	ICTLEU01	100	0		Migrant	M
Hepatic tanager (*Piranga flava*)	(ICTLEU01)	99	1	PIRFLA18 (GenBank OR760427)	Migrant	H
Western tanager (*Piranga ludoviciana*)	(ICTLEU01)	99	1	PIRFLA18 (GenBank OR760426)	Migrant	H
Hepatic tanager (*Piranga flava)*	(ICTLEU01)	99	3	PIRFLA19 (GenBank OR760377)	Migrant	M
Cassin's kingbird (*Tyrannus vociferans*)	(ICTLEU01)	99	1	TYRVOC05 (GenBank OR760410)	Migrant	M
Western tanager (*Piranga ludoviciana*) (2)	(LINOLI02)	98	7	PIRLUD21 (GenBank OR760362)	Migrant	H
Cassin's kingbird (*Tyrannus vociferans*)	(MYMAC01)	99	2	TYRVOC04 (GenBank OR760307)	Migrant	H
Western wood peewee (*Contopus sordidulus*)	MYMAC02	100	0		Migrant	H
Western wood peewee (*Contopus sordidulus*)	PACPEC02	100	0		Migrant	H
Western tanager (*Piranga ludoviciana*)	PACPEC02	100	0		Migrant	H
Hepatic tanager (*Piranga flava*) (2)	PACPEC02	100	0		Migrant	H
Western tanager (*Piranga ludoviciana*) (3)	(PACPEC02)	99	1	PIRLUD15 (GenBank OR760331, OR760332, OR760373)	Migrant	H
Hepatic tanager (*Piranga flava*)	(PACPEC02)	99	1	PIRLUD16 (GenBank OR760379)	Migrant	M
Western tanager (*Piranga ludoviciana*)	(PACPEC02)	99	1	PIRLUD16 (GenBank OR760372)	Migrant	H
Western tanager (*Piranga ludoviciana*)	(PACPEC02)	99	1	PIRLUD17 (GenBank OR760380)	Migrant	H
Western tanager (*Piranga ludoviciana*)	(PACPEC02)	99	2	PIRLUD18 (GenBank OR760386)	Migrant	H
Chipping sparrow (*Spizella passerina*)	PHEMEL02	100	0		Resident	H
Black-headed grosbeak (*Pheucticus melanocephalus*) (22)	PHEMEL02	100	0		Migrant	M, H
Black-headed grosbeak (*Pheucticus melanocephalus*) (3)	(PHEMEL02)	99	1	PHEMEL04 (GenBank OR760333, OR760421, OR760433)	Migrant	H
Black-headed grosbeak (*Pheucticus melanocephalus*)	(PHEMEL02)	99	1	PHEMEL05 (GenBank OR760382)	Migrant	H
Black-headed grosbeak (*Pheucticus melanocephalus*)	(PHEMEL02)	99	1	PHEMEL06 (GenBank OR760446)	Migrant	H
Black-headed grosbeak (*Pheucticus melanocephalus*)	(PHEMEL02)	99	1	PHEMEL07 (GenBank OR760388)	Migrant	H
Western tanager (*Piranga ludoviciana*)	(PIOLI04)	99	1	PIRLUD19 (GenBank OR760374)	Migrant	H
Western tanager (*Piranga ludoviciana*)	(PIOLI04)	99	2	PIRLUD20 (GenBank OR760375)	Migrant	H
Western tanager (*Piranga ludoviciana*)	(PIOLI04)	99	2	PIRLUD21 (GenBank OR760362)	Migrant	H
Spotted towhee (*Pipilo maculatus*)	PIPMAC01	100	0		Resident	H
Chipping sparrow (*Spizella passerina*) (2)	(PIPMAC03)	99	1	SPIPAS22 (GenBank OR760394, OR760440)	Resident	M
Chipping sparrow (*Spizella passerina*)	(PIPMAC03)	99	1	SPIPAS23 (GenBank OR760396)	Resident	M
Hepatic tanager (*Piranga flava*) (4)	PIRFLA01	100	0		Migrant	L, M
Western tanager (*Piranga ludoviciana*)	PIRFLA09	100	0		Migrant	H
Western tanager (*Piranga ludoviciana*)	(PIRFLA08)	97	13	PIRLUD11 (GenBank OR760365)	Migrant	H
Hepatic tanager (*Piranga flava*)	(PIRFLA10)	99	1	PIRFLA17 (GenBank OR760376)	Migrant	M
Western tanager (*Piranga ludoviciana*)	PIRLUD01	100	0		Migrant	H
Western tanager (*Piranga ludoviciana*) (2)	PIRLUD02	100	0		Migrant	H
Hepatic tanager (*Piranga flava*)	(PIRLUD08)	99	1	PIRFLA20 (GenBank OR760428)	Migrant	H
Western tanager (*Piranga ludoviciana*)	(PIRLUD08)	99	1	PIRFLA20 (GenBank OR760425)	Migrant	H
Willow flycatcher (*Empidonax traillii*)	(PIRLUD08)	99	1	PIRFLA20 (GenBank OR760444)	Migrant	H
Western tanager (*Piranga ludoviciana*) (2)	PIRLUD08	100	0		Migrant	M
Willow flycatcher (*Empidonax traillii*)	PIRLUD08	100	0		Migrant	H
Hepatic tanager (*Piranga flava*)	PIRLUD09	100	0		Migrant	H
Chipping sparrow (*Spizella passerina*)	(QUIQUI01)	98	10	SPIPAS19 (GenBank OR760405)	Resident	M
Chipping sparrow (*Spizella passerina*)	(SETAUD07)	97	13	SPIPAS20 (GenBank OR706406)	Resident	M
Chipping sparrow (*Spizella passerina*) (2)	SPIPAS01	100	0		Resident	H
Western tanager (*Piranga ludoviciana*)	(SPIPAS02)	94	25	PIRLUD13 (GenBank OR760364)	Migrant	H
Chipping sparrow (*Spizella passerina*)	(SPIPAS06)	98	10	SPIPAS21 (GenBank OR760404)	Resident	M
Lesser goldfinch (*Spinus psaltria*)	SPISAL02	100	0		Resident	H
Yellow-rumped warbler (*Setophaga coronata*)	TABI02	100	0		Resident	H
Chipping sparrow (Spizella passerina) (5)	TABI02	100	0		Resident	L, M, H
Black-chinned sparrow (*Spizella atrogularis*)	TABI02	100	0		Resident	H
Cassin's kingbird (*Tyrannus vociferans*)	TABI02	100	0		Migrant	H
Western bluebird (*Sialia mexicana*)	TABI02	100	0		Resident	H
Scott's oriole (*Icterus parisorum*)	TABI02	100	0		Migrant	H
Grace's warbler (*Setophaga graciae*)	TABI02	100	0		Migrant	H
Grace's warbler (*Setophaga graciae*)	(TABI02)	99	1	SETGRA04 (GenBank OR760419)	Migrant	H
Western bluebird (*Sialia mexicana*)	(TABI02)	96	14	SIAMEX06 (GenBank OR760429)	Resident	H
Violet-green swallow (*Tachycineta thalassina*)	TABI10	100	0		Migrant	M
Chipping sparrow (*Spizella passerina*)	(TURFUL01)	99	5	SPIPAS24 (GenBank OR760383)	Resident	H
Chipping sparrow (*Spizella passerina*)	(TURFUL01)	99	2	SPIPAS25 (GenBank OR760384)	Resident	H
Ash-throated flycatcher (*Myiarchus cinerascens*)	TOXCUR01	100	0		Migrant	M
Warbling vireo (*Vireo gilvus*)	VIGIL07	100	0		Migrant	H
Plumbeous vireo (*Vireo plumbeus*)	(VIOLI18)	99	4	VIRPLU10 (GenBank OR760310)	Migrant	M
Plumbeous vireo (*Vireo plumbeus*)	(VIRPLU07)	99	1	VIRPLU11 (GenBank OR760311)	Migrant	M
White-winged dove (*Zenaida asiatica*)	(ZEMAC12)	95	21	ZENASI01 (GenBank OR760399)	Resident	M
Mourning dove (*Zenaida macroura*)	ZEMAC13	100	0		Resident	M
Mourning dove (*Zenaida macroura*)	ZEMAC17	100	0		Resident	M
*Leucocytozoon*						
Western tanager (*Piranga ludoviciana*)	PIRFLA02	100	0		Migrant	H
Black-headed grosbeak (*Pheucticus melanocephalus*)	(PIRLUD04)	99	2	PHEMEL03 (GenBank OR760442)	Migrant	H
*Plasmodium*						
Lark sparrow (*Chondestes grammacus*)	EULNIG01	100	0		Migrant	H
Spotted towhee (*Pipilo maculatus*)	LAIRI01	100	0		Resident	H
Lark sparrow (*Chondestes grammacus*)	LAIRI01	100	0		Resident	M
Western bluebird (*Sialia mexicana*)	MOLATE01	100	0		Resident	M
Chipping sparrow (*Spizella passerina*)	(MOLATE01)	98	7	SPIPAS18 (GenBank OR760369)	Resident	H
Blue grosbeak (*Passerina caerulea*)	SEIAUR01	100	0		Migrant	M
Western tanager (*Piranga ludoviciana*)	(SEIAUR01)	99	2	PIRLUD12 (GenBank OR760367)	Migrant	H
Spotted towhee (*Pipilo maculatus*) (4)	SETCOR03	100	0		Resident	H
Hepatic tanager (*Piranga flava*)	SETCOR03	100	0		Migrant	H
Western tanager (*Piranga ludoviciana*)	SETCOR03	100	0		Migrant	H
Willow flycatcher (*Empidonax traillii*) (2)	SETCOR03	100	0		Migrant	L, M
Black-headed grosbeak (*Pheucticus melanocephalus*)	TACTHA01	100	0		Migrant	H
Western tanager (*Piranga ludoviciana*)	(TACTHA01)	99	4	PIRLUD14 (GenBank OR760366)	Migrant	H
Bewick's wren (*Thryomanes bewickii*)	TROAED24	100	0		Resident	H
Chipping sparrow (*Spizella passerina*)	VIOLI03	100	0		Resident	M

In column two, lineages without parentheses are 100% matches to lineages in the Malavi database (determined *via* MalAvi blast), and lineages in parentheses reflect closest lineage *via* MalAvi blast. For the latter lineages, we indicate the per cent match and base pair (BP) differences and provide a novel lineage designation per MalAvi protocols. Migratory status denotes whether a species if present year-round (resident) or is only present during the breeding season (migrant). Elevation refers to the elevation of our sampling locations, L signifies low elevation which we consider to be ~1700 m, M signifies medium elevation which we consider ~1800 m and H denotes high elevations ~2000 m. For both our low and medium elevations, 2 sites were pooled into the low elevation (1746 and 1769 m) and medium elevation (1852 and 1875 m). Although we have designated an elevation gradient of low, medium, and high is it important to note that the elevation gradient is of ~400 m.

Many of the novel lineages detected in this study are not highly distinct from the next closest lineage available on the MalAvi database. There were 15 novel lineages that differed from Malavi's closest match by 1 base pair, 8 novel lineages that differed by 2 to 5 base pairs, and 9 novel lineages that differed by 6 or more base pairs (Supplemental Fig. 1).

Regarding host breadth, we recovered 8 specialist lineages (8 *Haemoproteus*) and 21 generalist lineages (13 *Haemoproteus*, 1 *Leucocytozoon* and 7 *Plasmodium*) (Supplemental Table 1), according to the threshold established by Drovetski *et al*. (2014).

## Discussion

Avian haemosporidian infection prevalence can be influenced by climatic variables, life history and migratory status. Our aim was to elucidate haemosporidian communities in the Davis Mountains by sampling breeding birds in this understudied region. We hypothesized that *Haemoproteus* would have higher prevalence than other genera, migrants would display higher infection rates than residents, prevalence and number of lineages would be similar to New Mexico sky islands, and that novel lineages would be observed more frequently in resident species. Our findings demonstrate a host association with lineage prevalence and may explain parasite prevalence on sky islands in the American southwest.

### Overall detection and comparison to other nearby regions

Our overall haemosporidian detection rate was 40.8%, consistent with the breeding birds on sky islands in New Mexico (36.6–36.1%) (Marroquin-Flores *et al*., 2017; Barrow *et al*., 2021). Our detection rate was slightly higher than south Texas (25.69%) (Keith *et al*., 2022) and central Mexico (22%) (Ham-Dueñas *et al*., 2017) but lower than the Texas Gulf coast (48.4%) (DeBrock *et al*., 2021); however, the latter study sampled individuals at a stopover site for migrating Nearctic-Neotropical birds, which may explain the higher prevalence (Newton, 2007).

We hypothesized that *Haemoproteus* would be the most abundant genus followed by *Leucocytozoon* and then *Plasmodium*, similar to the abundance prevalence on the sky islands of New Mexico (Marroquin-Flores *et al*., 2017; Barrow *et al*., 2021). While we did find that *Haemoproteus* was the most abundant genus (88.9%), our study differed by finding *Plasmodium* (16.7%) to be more abundant than *Leucocytozoon* (1.9%). Our finding that *Haemoproteus* was most prevalent is not surprising because *Haemoproteus* is globally the most prevalent haemosporidian genera, and generally most prevalent at higher elevations and in arid environments (Clark *et al*., 2014; Gupta *et al*., 2019; Keith *et al*., 2022). It is possible that we found a greater abundance of *Plasmodium* than *Leucocytozoon* due to the elevation at which we conducted our sampling. Multiple studies have observed *Leucocytozoon* to be more prevalent at higher elevations and *Plasmodium* more prevalent at lower elevations (Van Rooyen *et al*., 2013; Illera *et al*., 2017; Fecchio *et al*., 2021). Our study sampled birds within an elevational band of ~1700–2200 m, which was lower than that of Barrow *et al*. (2021) and Marroquin-Flores *et al*. (2017), who conducted their New Mexico sky island sampling between elevations of 2100–2500 m.

### Prevalence by elevation, age, sex, foraging height and migration status

We found that haemosporidian prevalence was not influenced by elevation, consistent with the findings of González *et al*. (2014), which found no significant correlation between prevalence and elevation. This is likely due to the small elevational differences between our 2 best-sampled sites (Table 2). Studies that have found a correlation between prevalence and elevation typically have sampled across elevational gradients ranging from approximately 300–1300 m (Illera *et al*., 2017; Williamson *et al*., 2019; Gupta *et al*., 2020; Pellegrino *et al*., 2021; Lau *et al*., 2022).

We found higher haemosporidian prevalence in adult than hatch-year birds, adding to the body of literature that has found a positive association between age and haemosporidian prevalence in birds. This association with age has been observed in 22 avian species sampled in the Missouri Ozarks (Ellis *et al*., 2014), White-banded Tanagers in Brazil (Fecchio *et al*., 2015), and Blue Tits in Sweden (Podmokła *et al*., 2014) and the United Kingdom (Wood *et al*., 2007). However, Asghar *et al*. (2011) and Matthews *et al*. (2016) found no significant effect of age on infection in Great Reed Warblers in Sweden or 25 species sampled in Tenessee, respectively. Furthermore, Bosholn *et al*. (2020) and Van Oers *et al*. (2010) found that hatch-year individuals had higher haemosporidian prevalence in Blue-crowned Manakins in Brazil and Seychelles Warblers on the Cousin Island in the Seychelles. Our observed higher rate of parasite prevalence in older individuals may reflect downregulation of the immune response during reproduction (Deviche *et al*., 2001; Deviche and Parris, 2006), increased exposure of adults to vectors (e.g. from nesting activities or increased foraging during reproduction) (Zuk and McKean, 1996; McCurdy *et al*., 1998), or a higher mortality rate for infected juveniles (e.g. Van Oers *et al*., 2010) which results in a smaller proportion of infected juveniles. The fact that we did detect infected hatch-year individuals in this study suggests local transmission of *Plasmodium* and *Haemoproteus* in the Davis Mountains.

The abundance of *Haemoproteus* in the Davis Mountains suggests that *Haemoproteus* vectors may be more successful in this environment, possibly because their developmental requirements of freshwater are less rigid than vectors of *Plasmodium* and *Leukocytozoon* (mosquitos and *Simulium* black flies, respectively), which require standing or flowing water for development (Adler, 2005; Eldridge, 2005). However, due to the monsoon rains that occur during the breeding season, water availability is an unlikely limiting factor for dipteran vector development. We are unaware of studies that would confirm high abundance of *Haemoproteus* dipteran vectors in this region during summer months.

In this study, both male and female birds were equally likely to be infected by haemosporidians. Some previous studies have found sex differences in parasite infection (Zuk and McKean, 1996), with prevalence and density generally higher in males (Wood *et al*., 2007; Van Oers *et al*., 2010; Cornelius *et al*., 2014), but occasionally higher in females (Asghar *et al*., 2011). However, similar to our results, many other studies have found no sex differences in infection rates (McCurdy *et al*., 1998; Ricklefs *et al*., 2005; Fecchio *et al*., 2015; Matthews *et al*., 2016; Bosholn *et al*., 2020). For example, in a community survey of 25 species in Tennessee and 42 species in Missouri, infection status did not vary with sex (Ricklefs *et al*., 2005; Matthews *et al*., 2016). In our study, several species were monomorphic in plumage and were not included in the sex analysis. Thus, our limited sample size may have decreased our power to detect sex differences. However, given the large number of studies that similarly report a lack of sex differences in infection, it is probable that sex differences are species and/or region specific depending on species life history and risk factors.

Our data indicate that foraging behaviour did not influence haemosporidian infection for our study in the Davis Mountains. These results contrast with the findings of Gupta *et al*. (2020) that found higher *Plasmodium* prevalence in species foraging at the ground level compared to the canopy level on the Western Ghats Sky Islands. Similarly, our findings contrast with those of DeBrock *et al*. (2021) that found ground and understory foragers were more likely than canopy foragers to be infected with *Plasmodium,* while canopy foragers were more likely to be infected with *Haemoproteus* at a migratory stopover sight on the Texas Gulf coast. Finally, Astudillo *et al*. (2013) found birds foraging in the low-middle strata were more likely to be infected by *Haemoproteus* in Georgia. We suspect that our observed lack of association between haemosporidian lineage and foraging height was because most positive infections in our study were *Haemoproteu*s, perhaps due to environmental/insect vector constraints on other parasite lineages.

Finally, we found parasite prevalence to be independent of migratory status. Previous work has found a higher parasite prevalence in migratory than resident birds in South America (Anjos *et al*., 2021; de Angeli Dutra *et al*., 2021*a*, 2021*b*) due to their increased exposure to vectors and parasites (Waldenstrom *et al*., 2002; Jenkins *et al*., 2012). However, our results and those of other studies conducted within the United States and northern South America suggest that this is not always the case (Astudillo *et al*., 2013; Matthews *et al*., 2016; Ricklefs *et al*., 2017). Some studies have found prevalence to actually be higher in resident than migratory birds (Pellegrino *et al*., 2021; Keith *et al*., 2022). Thus, migratory status appears to have conflicting relationships with prevalence, and our results appear to reflect similar transmission rates between migrants and residents coexisting in the Davis Mountains.

### Lineage comparisons

We found support for our hypothesis that parasite lineages in the Davis Mountains would be similar to those detected on the sky islands of New Mexico. Of the previously described lineages that we detected, 71.8% (*n* = 23) were also detected in New Mexico according to the Malavi database. Lineage comparisons determined that the Davis Mountains shared more lineages with the New Mexico sky islands than with south Texas (18.7%), the Texas Gulf coast (21.9%) or central Mexico (0%) (Ham-Dueñas *et al*., 2017; DeBrock *et al*., 2021; Keith *et al*., 2022). This is likely due to the similarity in host communities and regional environmental variables between the Davis Mountains and New Mexico sky islands (Fecchio *et al*., 2019). Most of the shared lineages between the Davis Mountains and south Texas (CHOGRA01, ICTLEU01, LAIRI01, PIRLUD02, SEIAUR01 and ZEMAC17) and the Texas Gulf coast (PACPEC02, PHEMEL02, PIRLUD02, SEIAUR01, SETCOR03, VIGIL07, VIOLI03), were found in multiple host species. We likely did not find shared lineages between the Davis Mountains and central Mexico because in central Mexico only Black-throated Sparrows were sampled, a species that was not sampled in our study.

Regarding host breadth and haemosporidian genus, our results support those of Fallon *et al*. (2005) that found a higher proportion of specialist lineages in *Haemoproteus* than *Plasmodium*, and the findings of Ellis *et al*. (2020) that found *Plasmodium* lineages to be mostly host generalists. Although we hypothesized that we would find more specialist than generalist lineages in resident species due to the geographic isolation of the Davis Mountains, we detected more generalist lineages in residents and found no association between host breadth and migratory status. We observed similar numbers of specialized lineages in migrants (*n* = 5 lineages) as residents (*n* = 4 lineages). These results contrast with those of Jenkins *et al*. (2012) that found residents to harbour more specialized parasites than migrants. However, we did find that some parasites have specific associations with their avian hosts in this region, occurring only on certain species. It is likely that host phylogeny plays a more significant role in parasite prevalence than migratory status (Ricklefs and Fallon, 2002; Ricklefs *et al*., 2004; Medeiros *et al*., 2013; Scordato and Kardish, 2014; Ellis *et al*., 2015; Clark *et al*., 2017; Clark and Clegg, 2017; Pulgarín-R *et al*., 2018; Ellis *et al*., 2020).

We also found that 55% (*n* = 39) of our detected lineages were previously unreported, consistent with the results of Barrow *et al*. (2021) and Marroquin-Flores *et al*. (2017), which found high percentages of novel lineages (56 and 63% respectively) on New Mexico sky islands. Surveys in the sky islands of New Mexico and the Davis Mountains appear to detect a larger percentage of novel lineages than those in south Texas (43%; (Keith *et al*., 2022), the Texas Gulf coast (26%; (DeBrock *et al*., 2021) and central Mexico (25%; (Ham-Dueñas *et al*., 2017). We hypothesized that more novel lineages would be found in resident than migratory species. Although we did detect a trend toward more novel lineages in migratory species (*n* = 26) than residents (*n* = 13), this did not reach statistical significance. It is probable that the sky islands in the American southwest have high numbers of novel lineages due to limited sampling in these regions. Our study supports this interpretation as most of the novel lineages we found lacked significant diversification compared to previously described lineages. Ultimately, avian host phylogeny appears to have a greater influence on parasite prevalence and detection of novel lineages than migratory status of birds or geographic barriers in the American southwest (Williamson *et al*., 2019).

In conclusion, we found that age but not sex, elevation, or foraging height influenced parasite prevalence in the Davis Mountains, with adult birds more likely to be infected with haemosporidians than hatch-year birds. Similar to other studies on sky islands, we found *Haemoproteus* to be the most abundant haemosporidian genus. We also found a large number of novel lineages in the region, many of them in migrant species. Surprisingly, we did not find a significant difference in parasite prevalence between residents and migrants. It is likely that avian host composition drives the prevalence of specific lineages detected, followed by geographic and environmental ranges. Overall, this study highlights the importance of host–parasite relationships on haemosporidian parasite distribution, and the importance of regional studies contributing to the knowledge of haemosporidian distribution patterns and their effects on avian hosts. Future studies should investigate the influence of regional differences on host–parasite relationships.

## Supporting information

Martinez et al. supplementary material 1 Martinez et al. supplementary material

Martinez et al. supplementary material 2 Martinez et al. supplementary material

## Data Availability

All data that supports the findings of this study are available within the article and its supplementary materials.

## Supplementary material

The supplementary material for this article can be found at https://doi.org/10.1017/S0031182023001087
