# Supplementary figures and images for: Avian haemosporidians of breeding birds in the Davis Mountains sky-islands of west Texas, USA
Source: Parasitology. 2023 Nov 9;150(14):1266–76. doi: 10.1017/S0031182023001087 (PMC10941211; doi:10.1017/S0031182023001087)

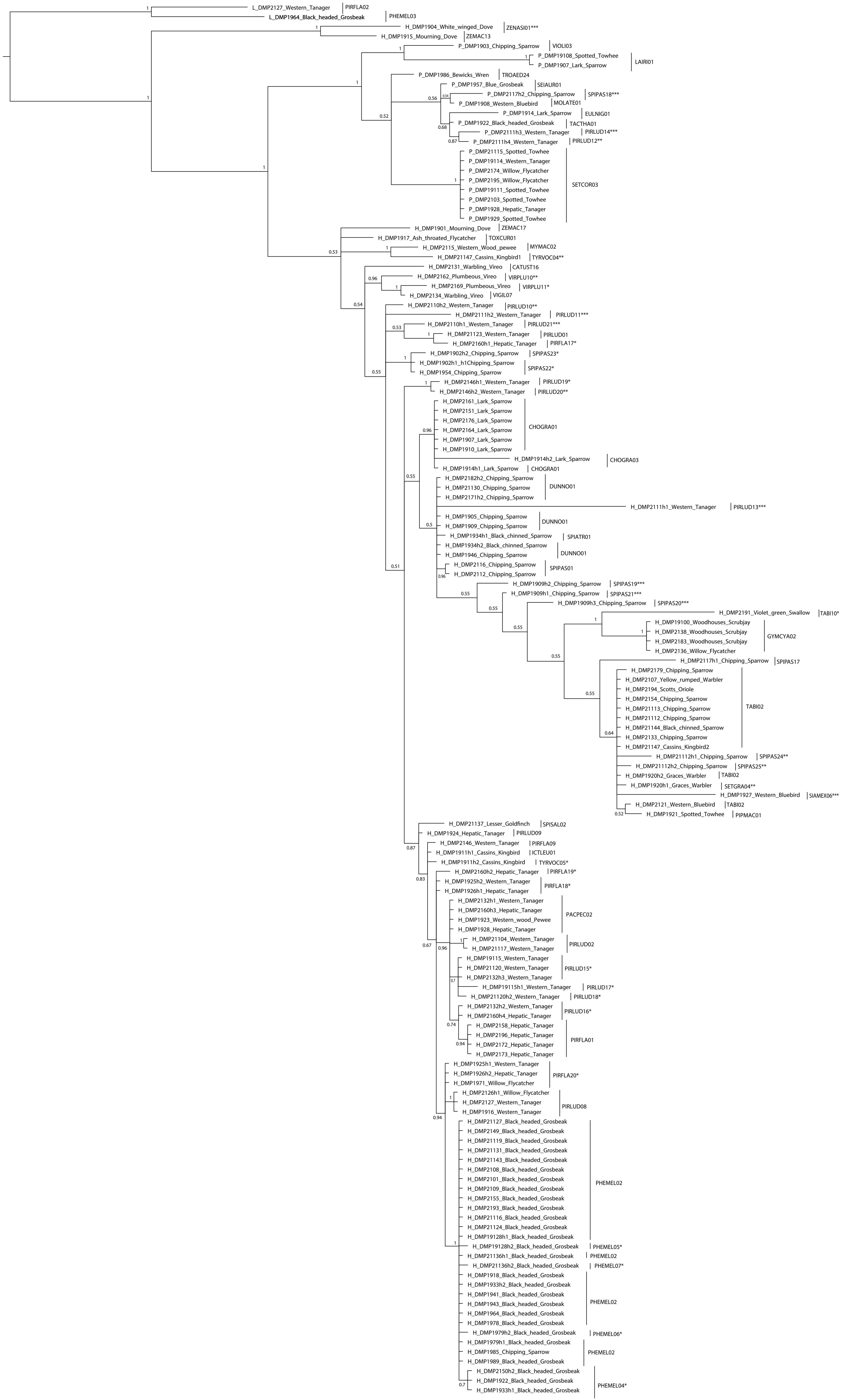

Supplement: Martinez et al. supplementary material 1 — Martinez et al. supplementary material [file S0031182023001087sup001.pdf]
