# Supplementary material for: Avian haemosporidians of breeding birds in the Davis Mountains sky-islands of west Texas, USA
Source: Parasitology. 2023 Nov 9;150(14):1266–76. doi: 10.1017/S0031182023001087 (PMC10941211; doi:10.1017/S0031182023001087)
Supplement: Martinez et al. supplementary material 2 — Martinez et al. supplementary material [file S0031182023001087sup002.docx]

**Supplemental Table 1.** Previously detected *Haemoproteus*, *Leucocytozoon*, and *Plasmodium* lineages found in the Davis Mountains Preserve.

| **Lineage Name** | **Host Genus in TX Study** | **Host Genus/Genera Previously Identified** | **Previous Locations Identified** |
| --- | --- | --- | --- |
| *Haemoproteus* |  |  |  |
| CATUST16 | *Vireo* | *Catharus, Dendroica, Vireo* | California, New Mexico |
| CHOGRA01 | *Chondestes* | *Chondestes* | New Mexico, Texas |
| DUNNO01 | *Spizella^a^* | *Carduelis, Emberiza, Ixoreus, Junco, Lagopus, Passerella, Prunella, Zonotrichia* | UK, Alaska, Sweden, Slovakia |
| GYMCYA02 | *Aphelocoma, Empidonax*^a^ | *Aphelocoma, Gymnorhinus* | New Mexico |
| ICTLEU01 | *Tyrannus^a^* | *Icterus, Coereba, Dolichonyx,* *Molothrus* | Nebraska, New Hampshire, Vermont |
| MYMAC02 | *Contopus*^a^ | *Myiodynastes* | Brazil^b^ |
| PACPEC02 | *Piranga* | *Amphispiza, Caryothraustes, Coereba, Euphonia, Hemitriccus,*  *Junco, Mionectes, Nemosia,*  *Pachycephala, Pipilo, Piranga, Saltator* | Brazil, Colombia, Ecuador, Guyana, Mexico,  Papua New Guinea,  United States |
| PHEMEL02 | *Pheucticus* | *Adelomyia, Chaetocercus, Cranioleuca, Empidonax,*  *Heliangelus, Lafresnaya, Molothrus, Ochthoeca,*  *Pheucticus, Synallaxis,*  *Tangara* | Peru, United States |
| PIPMAC01 | *Pipilo* | *Pipilo* | New Mexico |
| PIRFLA01 | *Piranga* | *Piranga, Spizella, Vireo* | New Mexico |
| PIRFLA09 | *Piranga* | *Piranga, Junco* | New Mexico |
| PIRLUD01 | *Piranga* | *Piranga* | New Mexico |
| PIRLUD02 | *Piranga* | *Euphonia, Hylocichla, Junco,*  *Microrhopias, Piranga, Setophaga* | New Mexico, Texas, Peru |
| PIRLUD08 | *Piranga* | *Piranga* | New Mexico |
| PIRLUD09 | *Piranga* | *Piranga* | New Mexico |
| PIRLUD01 | *Piranga* | *Piranga* | New Mexico |
| PIRLUD02 | *Piranga* | *Euphonia, Hylocichla, Junco,*  *Microrhopias, Piranga, Setophaga* | New Mexico, Texas, Peru |
| PIRLUD08 | *Piranga* | *Piranga* | New Mexico |
| PIRLUD09 | *Piranga* | *Piranga* | New Mexico |
| SPIPAS01 | *Spizella* | *Aphelocoma, Carduelis, Junco,*  *Loxia, Pipilo, Spizella* | Michigan, New Mexico |
| TABI02 | *Icterus*^a^*, Setophaga, Sialia*^a^*, Spizella*^a^*, Tyrannus*^a^*,* | *Amazilia, Carpodacus, Dendroica,*  *Elaenia, Geothlypis, Parula, Setophaga, Sporophila, Tachycineta* | Michigan, Missouri, New Mexico, New York, Peru |
| TABI10 | *Tachycineta* | *Tachycineta* | Michigan, New Mexico |
| TOXCUR01 | *Myiarchus* | NA | NA |
| VIGIL07 | *Vireo* | *Vireo* | California, Michigan, New Mexico, Texas |
| ZEMAC13 | *Zenaida* | *Zenaida* | Mexico |
| ZEMAC17 | *Zenaida* | NA | NA |
| *Leucocytozoon* |  |  |  |
| PIRFLA02 | *Piranga* | *Carduelis, Pheucticus, Piranga* | New Mexico |
| *Plasmodium* |  |  |  |
| EULNIG01 | *Chondestes*^a^ | *Eulacestoma, Icteria* | Papua New Guinea, United States |
| LAIRI01 | *Pipilo*^a^ | *Amphispiza, Anas, Aphelocoma,*  *Baeolophus, Carduelis, Catharus, Colinus, Corvus, Dendroica,*  *Dolichonyx, Geospiza, Gymnorhinus, Lamprotornis,*  *Loxia, Passerculus, Poecile,*  *Sialia, Strix, Tachycineta,*  *Thryomanes, Troglodytes, Turdus, Tyrannus* | Alaska, Alberta, British Columbia, California,  Galapagos, New Hampshire, New Mexico |
| MOLATE01 | *Sialia* | *Carpodacus, Molothrus, Pipilo, Baeolophus, Spizella, Loxia, Sialia* | California, Idaho, New Mexico, Mexico |
|  |  |  |  |
| SEIAUR01 | *Passerina*^a^ | *Agelaius, Ammodramus, Aphelocoma, Cardinalis,*  *Carpodacus, Catharus,*  *Cyanocorax, Dendroica, Dolichonyx, Geospizopsis,*  *Junco, Larosterna, Megascops,*  *Melospiza, Molothrus, Parus,*  *Passer, Pheucticus, Pipilo,*  *Poecile, Salpinctes, Seiurus,*  *Serinus, Setophaga, Somateria, Spiza, Spizella, Strix, Tachycineta, Thryomanes, Turdus, Tyto, Zonotrichia* | Alaska, Alberta, Argentina, Arizona  Bermuda, California,  Colombia, Colorado,  Florida, Georgia, Kentucky, Mexico,  Michigan, Missouri, Nebraska, New Mexico,  New York, Oregon, Pennsylvania, Quebec,  Texas, Vermont, Wisconsin |
|  |  |  |  |
| SETCOR03 | *Pipilo*^a^*, Piranga*^a^*, Empidonax*^a^ | *Dendroica, Setophaga, Vireo* | British Columbia, Texas |
| TACTHA01 | *Pheucticus^a^* | *Tachycineta, Piranga* | New Mexico, Texas |
| TROAED24 | *Thryomanes* | *Amphispiza, Aphelocoma,*  *Gymnorhinus, Junco, Poecile, Polioptila, Sialia, Spizella, Thryomanes, Troglodytes* | California, Colorado, New Mexico, Mexico |
| VIOLI03 | *Spizella*^a^ | *Troglodytes,*  *Vireo,*  *Mionectes,*  *Monasa,*  *Clytolaema* | Brazil, Peru, United States |

^a^ Novel association between avian family and lineage

^b^ Novel association between geographic region and lineage

NA – information not available on MalAvi database

**Supplemental Figure 1.** Bayesian analysis of haemosporidians sampled in the Davis Mountains Preserve. One asterisk indicates the sequence had a 99% similarity with the closest match on Malavi, two asterisks indicate a two – five base pair difference, and three asterisks indicates a base pair difference of more than six. All lineages that had 99% or less similarity with their closest match on Malavi were designated new lineages.
